# Proactive and reactive inhibitory control in eating disorders

**DOI:** 10.1016/j.psychres.2017.06.073

**Published:** 2017-09

**Authors:** Savani Bartholdy, Samantha J. Rennalls, Claire Jacques, Hollie Danby, Iain C. Campbell, Ulrike Schmidt, Owen G. O’Daly

**Affiliations:** a Section of Eating Disorders, Department of Psychological Medicine, Institute of Psychiatry, Psychology & Neuroscience, King's College London, UK; b Department of Neuroimaging, Institute of Psychiatry, Psychology & Neuroscience, King's College London, UK; c Department of Neuroscience, Institute of Psychiatry, Psychology & Neuroscience, King's College London, UK

## Abstract

Altered inhibitory control has been implicated in the development and maintenance of eating disorders (ED), however it is unclear how different types of inhibitory control are affected across the EDs. We explored whether individuals with bulimia nervosa (BN), binge eating disorder (BED) and anorexia nervosa (AN) differed from healthy individuals (HC) on two types of motor inhibitory control: proactive inhibition (related to the preparation/initiation of a response) and reactive inhibition (withholding a response in reaction to a signal). Ninety-four women (28 AN, 27 BN, 11 BED, 28 HC) completed two neuropsychological tasks (a cued reaction time task and a stop signal task), and questionnaires assessing clinical variables, mood, anxiety, and inhibitory control. Self-reported inhibitory control was poorer in women with BN compared to the HC and AN groups, but greater in women with AN compared to all other groups. However, no group differences in reactive inhibition were observed. Proactive inhibition was augmented in women with AN compared to HC, and this was related to self-reported intolerance of uncertainty. The findings suggest that proactive inhibition may be a relevant target for behavioural interventions for AN, and call for further research into the relationship between intolerance of uncertainty and proactive inhibition.

## Highlights

• Women with eating disorders self-report altered motor inhibition.
• No behavioural differences in reactive inhibition on the stop signal task were observed.
• Proactive inhibition is elevated in adult women with anorexia nervosa.
• Group differences in proactive inhibition are related to intolerance of uncertainty.

## 1 Introduction

Altered inhibitory control, i.e., the ability to appropriately withhold a response (Bartholdy et al., 2016a, Wardak et al., 2012), has been implicated in the development and maintenance of eating disorders (EDs). For example, binge eating (a core symptom in bulimia nervosa [BN] and binge eating disorder [BED]) includes a perceived loss of control over eating. Studies report that individuals with BN or BED have poorer motor (Galimberti et al., 2012, Wu et al., 2013a) and reward-based (Kekic et al., 2016, Manasse et al., 2015) inhibitory control, i.e., the ability to inhibit an inappropriate motor response, and the ability to delay gratification, respectively. A meta-analysis revealed that individuals with bulimic-type EDs (BN, BED and anorexia nervosa (AN) binge-purge subtype) showed a general deficit (of small effect size) in inhibitory control compared to healthy controls (HC) using a range of neuropsychological tasks, with a greater deficit to disorder-relevant stimuli observed in individuals with BN (Wu et al., 2013b). In contrast, adult women with anorexia nervosa (AN), particularly individuals with AN restrictive subtype, have been reported to exhibit excessive reward-related inhibitory control compared to healthy individuals (Steinglass et al., 2012). However, these findings are not always consistent (for reviews, see Bartholdy et al., 2016c; McClelland et al., 2016).

EDs have been modelled along a spectrum of inhibitory control, with AN at the over-controlling end and BED at the opposite (impulsive) extremity (Brooks et al., 2012). However, it remains unclear whether different types of inhibitory control are similarly affected across the EDs, or whether any differences are disorder- or context-specific (Bartholdy et al., 2016c). For example, the evidence for such models with respect to reactive motor inhibitory control (i.e., withholding a response in reaction to a cue) is inconsistent (Bartholdy et al., 2016c). Moreover, there has been limited exploration of proactive motor inhibitory control, i.e., a form of inhibitory control related to the preparation or initiation of a response (Meyer and Bucci, 2016). When presented with a stimulus, one must decide whether a response is required, and if so, which response is appropriate. Proactive inhibitory control may involve adjusting one's reaction time (e.g., post-error slowing to improve accuracy), or a more general withholding of a response (i.e., taking longer to respond) when the need for a response is uncertain (see Bartholdy et al., 2016a for further description). As proactive inhibition may relate to ambiguity and uncertainty in the requirement for a response, it is possible that the degree to which proactive inhibition is exercised is related to one's tolerance of uncertainty, the degree of evidence required for a response to be provided, and one's priorities regarding performance (e.g., speed/accuracy). In the context of EDs, it has been hypothesised that proactive inhibition will manifest more strongly in individuals with AN, given the evidence for high perfectionism and intolerance of uncertainty in this population (Bartholdy et al., 2016a). However, proactive inhibition has not yet been explicitly explored or compared across the EDs. It is of note that the terms “impulsivity” and “inhibitory control” are often used to describe opposite patterns of behavioural responding, as they will be here for comparability with the existing literature, however these are distinct yet related constructs that should not be equated (see Bartholdy et al., 2016c for further discussion).

These types of motor inhibitory control may be relevant to the way in which symptoms are manifested in EDs and how decisions are made across contexts. For example, poor reactive inhibitory control may contribute to impulsive behaviours that reflect an inability to resist temptation or overcome urges, e.g., binge eating (Svaldi et al., 2014). In contrast, proactive inhibition may be related to decision making in the context of uncertainty, e.g., individuals may delay responses until further evidence has been gathered (prioritising accuracy over speed of responding), or jump to conclusions to reduce the time in which uncertainty is experienced (Bartholdy et al., 2016a). Individuals with EDs (particularly AN) are highly intolerant of uncertainty (Sternheim et al., 2011b) and studies have revealed intolerance of uncertainty to be positively related to symptom severity (Sternheim et al., 2011a, Sternheim et al., 2015). On this basis, it is possible that individuals with AN may exercise proactive inhibition more strongly in the context of uncertainty compared to HC, and that this may underscore their use of more inhibitory, restrictive and/or avoidant behavioural coping strategies.

This study compares proactive and reactive motor inhibitory control in individuals with AN, BN and BED to healthy controls (HC) and explores how proactive and reactive inhibition are related to ED pathology, anxiety and intolerance of uncertainty. We predict that participants with BN and BED will show less motor inhibitory control and participants with AN will show greater inhibitory control compared to HC. Poorer inhibitory control will be reflected by faster reaction times (RTs) in the context of uncertainty, poorer stop accuracy, and less benefit from warning cues. We also predict that poorer inhibitory control will be related to frequency of impulsive ED symptoms, i.e., binge eating and purging, and that inhibitory control (particularly proactive inhibition) will be related to intolerance of uncertainty.

## 2 Methods

### 2.1 Subjects

Ninety-four adult women participated: 28 with AN, 27 with BN, 11 with BED, and 30 healthy normal-weight women (HC). Healthy participants were recruited via online and poster advertisements at King's College London (KCL). Participants with an ED were recruited via online and poster advertisements at KCL, via the Eating Disorder Unit at the South London and Maudsley NHS Foundation Trust, via online adverts on the Beat website (the UK's national ED charity), and a research recruitment website (www.callforparticipants.com). ED diagnoses were confirmed using the Eating Disorder Diagnostic Scale (EDDS; Stice et al., 2000) and the screening module of the Structured Clinical Interview for DSM-IV Disorders, Researcher Version (SCID-IV; First et al., 2002). Participants in the ED groups were required to meet one of the following criteria: (a) DSM-5 diagnosis of AN and a body mass index (BMI) ≤ 18.5 kg/m^2^, (b) DSM-5 diagnosis of BN and a BMI > 18.5 kg/m^2^, or (c) DSM-5 diagnosis of BED and a BMI > 18.5 kg/m^2^. Participants taking psychotropic medication were included provided their medication had been stable for ≥ 14 days prior to the study. HC were required to be of healthy BMI (18.5–25 kg/m^2^) and never had an ED or any current/previous neurological/psychiatric disorder. Participants were excluded if they were using illicit drugs or were pregnant.

### 2.2 Computer tasks

#### 2.2.1 Cued reaction time (RT) task

This task was adapted from studies assessing proactive inhibition in individuals with Parkinson's disease (Ballanger et al., 2009, Boulinguez et al., 2009) and healthy individuals (Bartholdy et al., 2016b). A fixation cross, flanked by two empty boxes, was continuously presented on the screen. On each trial, a visual target (large yellow dot) appeared in one of the two boxes. Participants were instructed to indicate the location of the target by pressing the corresponding arrow key as quickly as possible. On some trials, the target was preceded by a spatially uninformative warning cue (the outline of the target appearing in both flanking boxes simultaneously; “cued trials”). This cue was intended to reduce uncertainty regarding the requirement of a response, but gave no information regarding what motor response would be required: participants were informed that the presence of the cue was warning them that the target would soon be appearing, and were reminded to only respond to the target and not the cue. The stimulus onset asynchrony (SOA) was manipulated so that the cue-target delay varied randomly across four conditions: 0 (no cue), 100, 300 and 500 ms. (See Supplement A for a schematic illustration of the task).

This study employed a blocked design (three experimental blocks and a practice block). The practice block included 18 trials (7 non-cued trials (0 ms SOA), 3 trials at 100 ms SOA, 4 trials at 300 ms SOA and 4 trials at 500 ms SOA). One experimental block consisted of only non-cued trials (“pure” block) whereas the other two included a mixture of cued and non-cued trials (“mixed” block), presented in a pseudorandomised order (the order of cued and non-cued trials was randomised but all participants received the same playlist order). The order in which the experimental blocks were completed was randomised and counterbalanced across participants. This task assessed proactive inhibition in two ways. (1) “Preparation cost” reflects the extent to which one's responses are slowed when not all stimuli are targets for response (Chikazoe et al., 2009). This is calculated by comparing the reaction times on all trials in the pure block (i.e., “certain” go trials) to non-cued (0 ms SOA) trials in the mixed block (i.e., “uncertain” go trials as the first visual stimulus on each trial may be either a target or a warning cue). (2) “Warning benefit” is the degree to which RT on cued trials benefit from longer SOAs (i.e., longer time to register the information provided by the warning cue: the next stimulus will be a target and therefore a response will certainly be required), calculated by comparing RTs on trials at each of the four SOAs (0 ms, 100 ms, 300 ms, 500 ms). These calculations will provide a more global assessment of proactive inhibition, indicating the degree to which proactive inhibition is more engaged at varying levels of certainty that a response is required. In order to assess the relationship between proactive inhibition and clinical variables, an index of preparation cost and warning benefit will be calculated by subtracting the mean reaction times in the pure block and the 500 ms SOA trials of the mixed blocks from the non-cued trials of the mixed blocks, respectively.

#### 2.2.2 Stop signal task (SST)

Participants were asked to provide rapid responses to a stimulus but to withhold their response in the presence of a stop signal. On each trial, participants were presented with a blue arrow (target) pointing left or right, and were asked to indicate the arrow's direction as quickly as possible using the keyboard's arrow keys. On a specified number of trials, a stop signal (red dot) was presented at a variable delay after the target. Participants were instructed to inhibit their response to the target if the stop signal appeared, but to not wait for the stop signal. The stop signal was presented at irregular intervals within each block to minimise predictability. The delay between the go signal and stop signal is termed the stop signal delay (SSD). The ability to inhibit a response is dependent on the length of the SSD: the longer the delay, the harder it is to inhibit a response. The SSD was varied from trial to trial in a staircase procedure (in 50 ms increments/decrements, minimum SSD set to 150 ms) that converged subjects toward an overall performance of 50% for each run. (See Supplement B for a schematic illustration of the task).

A blocked design was used, including a practice block (22 trials [of which 7 were stop trials]) and 4 experimental blocks that varied in the proportion of stop signal trials (0% 60 trials [0 stop trials]), 15% (200 trials [30 stop trials]), 25% (120 trials [30 stop trials]), 35% (86 trials [30 stop trials]). The block comprised of 25% stop trials was used to assess reactive inhibition, consistent with previous literature (Verbruggen et al., 2008). Differences in SST outcomes across the blocks were used to explore proactive inhibition (difference between the 35% and 15% blocks). The main outcome measures included mean RT, mean SSD, mean go accuracy, mean stop accuracy, post-error slowing, and stop signal reaction time (SSRT). The SSRT is considered an index of inhibitory control ability and is usually calculated by subtracting the SSD that enables convergence at 50% accurate inhibition from the mean RT on go trials. However, due to the variability in stop accuracy across the blocks and the small number of trials in our design, SSRT was calculated by subtracting the mean SSD on stop trials from the nth percentile of the correct go RT distribution, using the formula: SSRT = RT(m)-SSD, where m = n(number of correct go responses) * probability_(responding|signal)_ (Bartholdy et al., 2016b, Nederkoorn et al., 2012). Therefore, if participants correctly stopped on 25% of the trials, the mean SSD was subtracted from the 25th% RT on correct go trials (0.25*n_(correct go trials)_) to calculate the SSRT.

### 2.3 Procedure

Diagnosis and eligibility was confirmed through a telephone screen, including the EDDS, SCID, and a study-specific inclusion and exclusion screening questionnaire developed by us. The EDDS (Stice et al., 2000) is a brief validated self-report questionnaire assessing the presence and frequency/severity of symptoms within the preceding 3 months. This questionnaire was employed to confirm self-reported diagnoses in the ED groups, while placing minimal burden on the participants during the telephone screening. Eligible participants were invited to attend a single research visit. During this visit, participants completed a questionnaire battery including measures assessing clinical characteristics (Eating Disorder Examination Questionnaire (EDEQ)), hunger (via 10 cm visual analogue scales and open questions regarding time since last eaten, expected time of next meal), anxiety (Depression, Anxiety and Stress Scale (DASS-21)), Intolerance of Uncertainty Scale (IUS), and self-reported impulsivity (Barratt Impulsiveness Scale (BIS-11), Behavioural Inhibition Scale – Behavioural Activation Scale (BIS-BAS)). Participants then completed two computer tasks probing inhibitory control (cued RT task, SST). Finally, height and weight were measured. Participants were debriefed and compensated up to £15 for their time and travel.

### 2.4 Data analysis

Data were collected using in-house software and analysed using IBM^®^ SPSS^®^ software (Version 22). All tests were two-tailed and the level of significance was set at α=0.05. On the stop signal task, SSRT and mean RT (assessing reactive inhibition) were not normally distributed. Difference scores (between the blocks with the highest and lowest proportion of stop trials) for stop accuracy and post-error slowing were normally distributed, but not for go accuracy or mean RT. Logarithmic transformations normalised the distribution of the SSRT data, but not the mean RT data. Scores on the BIS-11 Attention and Motor subscales and DASS-21 Stress subscale were normally distributed, but those for all other subscales were not. Age, ED pathology (EDE-Q global score), binge frequency and hunger were not normally distributed.

One-way analyses of variance (ANOVAs) were conducted to assess the effect of group on self-reported and task-based inhibitory control. Where data were not normally distributed, Kruskal-Wallis tests were employed to explore the effect of group, and Friedman's tests were conducted to explore the effects of within-subject variables. Significant main effects were further explored using Bonferroni-corrected post-hoc *t*-tests and Mann-Whitney *U* tests. For ease of readability, we have reported all statistical results from post-hoc tests in a supplementary file (Supplement C). Given the *a priori* predictions of a relationship between inhibitory control and intolerance of uncertainty, a set of exploratory post-hoc analyses of covariance (ANCOVAs) were conducted to explore whether the main ANOVA results were influenced by confounding effects of intolerance of uncertainty. Group differences in specific clinical variables (length of illness, binge frequency and purge frequency) were compared across only the ED groups, whereas group comparisons on all other variables (including global ED pathology) included all 4 groups. Finally, Pearson and Spearman's correlations were conducted to explore the relationship between inhibitory control during the tasks, with respect to (a) task-based measures of proactive and reactive inhibitory control, (b) mood and anxiety (depression, anxiety, stress, intolerance of uncertainty); (c) self-reported impulsivity and (d) clinical variables (length of illness, global ED pathology (EDEQ global score), BMI, symptom frequency).

### 2.5 Ethics

Ethical approval was obtained from the London Bloomsbury NHS Research Ethics Committee (REC ref: 15/LO/0251). All participants gave written consent after the procedures were explained and were debriefed after the experiment.

## 3 Results

Ninety-four participants were included in the analysis. Due to computer malfunctions, incomplete data were collected from 3 participants (1 HC, 1 AN, 1 BN) on the SST though all completed the playlist with 25% stop trial and thus, these participants were excluded from comparisons across playlists (to explore proactive inhibition) but were included in assessments of reactive inhibition (using the data from the 25% stop trials playlist). A further 4 participants (1 HC, 1 AN, 2 BN) had very low levels of stop accuracy across all 4 blocks of the SST, indicating that the task was not completed correctly. These participants were excluded from all analyses of SST data. All participants completed the proactive inhibition task. Demographic and clinical information for each group is reported in Table 1. The groups did not differ in age, but did differ in global ED pathology (EDE-Q Global, driven by lower ED pathology in the HC group compared to all other groups) and BMI (driven by lower BMI in the AN group compared to all other groups and higher BMI in the BED group compared with the HC group). The ED groups differed in illness duration, with the greatest duration reported by the AN group and the shortest reported by the BED group. Amongst those who endorsed the behaviours, no differences in binge or purge frequency were reported across the ED groups. Finally, some individuals in the ED groups reported taking psychotropic medication, mostly antidepressants (AN: 39.2%, BN: 18.5%, BED: 9.1%). None of the participants reported taking benzodiazepines. One participant in the AN group was taking a low daily dose (75 mg) of quetiapine. Further information regarding the medication being taken by the present sample is reported in Bartholdy et al. (2017).

**Table 1:** Demographic and clinical information.

	HC (n = 28)	AN (n = 28)	BN (n = 27)	BED (n = 11)	Statistical evaluation of group differences^a^
**Demographic information** (mean [SD])					
Age (years)	24.64 (5.14)	30.00 (10.51)	25.30 (6.85)	28.73 (11.33)	χ^2^(3) = 4.590, *p* = 0.204
BMI (kg/m^2^)	22.04 (2.03)	16.55 (1.79)	22.57 (3.30)	28.86 (6.92)	*F*(3, 90) = 37.081, *p* < 0.001
Right handed (%)	82.14%	78.57%	96.30%	81.81%	
% completed undergraduate degree	100.00%	77.78%	79.17%	88.89%	
**ED-related variables** (mean [SD])					
EDE-Q Global	0.53 (0.66)	3.80 (1.29)	4.29 (1.00)	3.65 (1.08)	χ^*2*^(3) = 57.166, *p* < 0.001
Self-reported length of illness (years)	–	10.59 (10.04)	5 (5.51)	2.32 (3.99)	χ^2^(2) = 14.163, *p* = 0.001
Lowest weight at current height (kg)	–	35.81 (9.31)	49.78 (7.08)	62.55 (15.83)	
Binge eating episodes (n reported in preceding 28 days [mean frequency [SD])^b^	0	8	27	11	χ^*2*^(2) = 2.466, *p* = 0.291
	(0.00 [0.000])	(11.14 [22.34])	(18.04 [22.78])	(8.00 [5.15])	
Purge frequency^c^ (n reported in preceding 28 days [mean frequency [SD])	0	12	25	0	*t*(11.494) = 1.724, *p* = 0.111
	(0.00 [0.000])	(35.93 [83.97])	(24.65 [24.60])	(0.00 [0.000])	
*Self-induced vomiting episodes (n reported in preceding 28 days [mean frequency [SD])*	0	9	19	0	
	(0.00 [0.000])	(28.19 [82.78])	(16.94 [20.48])	(0.00 [0.00])	
*Laxative use (n reported in preceding 28 days [mean frequency [SD])*	0	5	12	0	
	(0.00 [0.000])	(9.07 [28.20])	(6.00 [9.90])	(0.00 [0.00])	
*Diuretic use (n reported in preceding 28 days [mean frequency [SD])*	0	0	4	0	
	(0.00 [0.000])	(0.00 [0.00])	(1.70 [5.60])	(0.00 [0.00])	
*n* reporting comorbid diagnoses (%)	0 (0%)	15 (53.2%)	9 (33.3%)	2 (18.2%)	
*n* currently taking medication^d^ (%, *n* taking antidepressants)	0 (0%)	19 (67.9%, 11)	8 (29.6%, 5)	5 (45.5%, 1)	
**Hunger** (mean [SD])					
VAS scale	0.31 (0.25)	0.20 (0.23)	0.28 (0.28)	0.40 (0.31)	χ^*2*^(3) = 6.065, *p* = 0.109

Re-printed and adapted with permission from Bartholdy et al. (2017), Temporal discounting in eating disorders, Eur. Eat. Disord. Rev., 2017, https://dx.doi.org/10.1002/erv.2513.

a Group differences were evaluated using Kruskal-Wallis chi-square tests, one-way ANOVAs and *t*-tests as appropriate.

b Comparison between those who reported binge eating in the AN, BN and BED groups.

c Comparison between those who reported any purging behaviour (including self-induced vomiting, laxative use or diuretic use) in the AN and BN groups.

d Not including oral or internal contraceptive medication.

### 3.1 Group differences in self-reported anxiety and mood

Self-reported depression, anxiety, stress and intolerance of uncertainty differed across the groups (all *p* < 0.001). This was driven by lower anxiety, stress, depression and intolerance of uncertainty in the HC group compared to all ED groups, with no differences on any measure observed between the ED groups (Table 2).

**Table 2:** Means (SDs) and comparisons between groups for self-reported depression, anxiety, stress and intolerance of uncertainty.

	Means (SD)				Kruskal-Wallis	Post-hoc Mann-Whitney *U* tests					
	HC	AN	BN	BED		HC vs. AN	HC vs. BN	HC vs. BED	AN vs. BN	AN vs. BED	BN vs. BED
DASS-21 Total	5.96 (5.687)	28.33 (15.350)	29.64 (15.250)	26.82 (16.582)	χ2(3) = 40.605, *p* < 0.001	*U* = 73.50, *z*= −5.042, *p* < 0.001*	*U* = 37.50, *z* = −5.504, *p* < 0.001*	*U* = 19.00, *z* = −4.181, *p* < 0.001*	*U* = 315.50, *z* = −0.404, *p* = 0.687	*U* = 140.00, *z*=−0.274, *p* = 0.784	*U* = 119.00, *z* = −0.636, *p* = 0.525
DASS-21 Depression	2.44 (4.449)	20.81 (13.579)	18.40 (12.288)	18.91 (11.811)	χ2(3) = 38.359, *p* < 0.001	*U* = 76.00, *z* = −5.066, *p* < 0.001*	*U* = 64.00, *z* = −5.079, *p* < 0.001*	*U* = 12.50, *z* = −4.488, *p* < 0.001*	*U* = 296.50, *z* = −0.753, *p* = 0.452	*U* = 136.50, *z* = −0.387, *p* = 0.699	*U* = 136.00, *z* = −0.052, *p* = 0.959
DASS-21 Anxiety	2.81 (3.813)	13.04 (11.071)	16.88 (10.818)	13.82 (10.971)	χ2(3) = 31.976, *p* < 0.001	*U* = 136.00, *z* = −4.028, *p* < 0.001*	*U* = 61.50, *z* = −5.113, *p* < 0.001*	*U* = 33.00, *z* = −3.810, *p* < 0.001*	*U* = 262.00, *z* = −1.386, *p* = 0.166	*U* = 137.50, *z* = −0.355, *p* 0.723	*U* = 114.00, *z* = −0.810, *p* = 0.418
DASS-21 Stress	6.67 (5.349)	22.81 (10.674)	24.00 (10.817)	20.91 (11.674)	χ2(3) = 35.553, *p* < 0.001	*U* = 82.50, *z*=−4.894, *p* < 0.001*	*U* = 58.50, *z* = −5.129, *p* < 0.001*	*U* = 39.00, *z* = −3.553, *p* < 0.001*	*U* = 312.00, *z* = −0.468, *p* = 0.640	*U* = 131.50, *z* = −0.549, *p* = 0.583	*U* = 113.00, *z* = −0.843, *p* = 0.399
IUS Total	43.78 (14.825)	81.52 (30.195)	79.54 (27.904)	76.82 (21.231)	χ2(3) = 32.238, *p* < 0.001	*U* = 88.50, *z* = −4.777, *p* < 0.001*	*U* = 92.00, *z* = −4.498, *p* < 0.001*	*U* = 27.00, *z* = −3.914, *p* < 0.001*	*U* = 318.50, *z* = −0.348, *p* = 0.728	*U* = 127.00, *z*=−0.692, *p* = 0.489	*U* = 128.50, *z* = −0.309, *p* = 0.757
IUS Uncertainty has negative behavioural and self-referent implications subscale (Self-Implications)	19.74 (5.868)	37.26 (14.730)	37.50 (13.215)	34.55 (11.750)	χ2(3) = 31.020, *p* < 0.001	*U* = 92.50, *z* = −4.710, *p* < 0.001*	*U* = 92.50, *z* = −4.495, *p* < 0.001*	*U* = 35.50, *z* = −3.645, *p* < 0.001*	*U* = 335.50, *z* = −0.037, *p* = 0.971	*U* = 122.00, *z* = −0.854, *p* = 0.393	*U* = 126.00, *z* = −0.395, *p* = 0.693
IUS Uncertainty about the future is unfair and spoils everything (Unfair Spoils)	22.11 (6.600)	38.70 (14.147)	36.56 (13.042)	37.82 (10.078)	χ2(3) = 28.797, *p* < 0.001	*U* = 101.50, *z* = −4.554, *p* < 0.001*	*U* = 118.00, *z* = −4.025, *p* < 0.001*	*U* = 29.00, *z* = −3.852, *p* < 0.001*	*U* = 288.00, *z* = −0.907, *p* = 0.364	*U* = 124.50, *z* = −0.774, *p* = 0.439	*U* = 131.00, *z* = −0.224, *p* = 0.823

* *p* < 0.05 after Bonferroni correction. Uncorrected *p*-values are presented in the table.

### 3.2 Cued RT task

Proactive inhibition was assessed using a simple cued RT paradigm. Participants, on average, responded fastest when the target followed the presentation of a cue after a 500 ms delay (Table 3). RTs were slowest during non-cued trials occurring in blocks containing a mixture of cued and non-cued trials.

**Table 3:** Mean (SD) reaction time and accuracy in the pure (non-cued trials only) and mixed (mixture of cued and non-cued trials) blocks of the simple cued reaction time paradigm.

	SOA	HC	AN	BN	BED
*Reaction time (ms)*					
	Pure	348.98 (55.10)	365.65 (70.75)	367.97 (72.58)	339.91 (45.83)
	0 ms	393.22 (51.24)	426.18 (85.42)	411.04 (52.12)	408.15 (55.44)
	100 ms	363.08 (48.61)	399.74 (76.36)	372.23 (46.07)	365.67 (43.81)
	300 ms	375.41 (48.38)	420.30 (82.94)	385.75 (47.66)	376.47 (56.49)
	500 ms	324.37 (47.98)	363.79 (82.21)	338.55 (58.56)	331.42 (38.62)
*Accuracy (%)*					
	Pure	98.8% (1.4%)	99.1% (1.6%)	97.8% (6.8%)	98.2% (2.5%)
	0 ms	99.6% (1.1%)	98.1% (5.6%)	99.6% (1.1%)	99.5% (1.0%)
	100 ms	97.7% (2.9%)	98.2% (2.8%)	96.3% (4.1%)	98.6% (2.3%)
	300 ms	97.7% (5.2%)	98.4% (2.7%)	98.0% (3.5%)	98.6% (2.3%)
	500 ms	96.6% (5.1%)	99.3% (1.8%)	98.0% (4.0%)	97.3% (5.2%)

#### 3.2.1 Preparation cost

Preparation cost was assessed by comparing mean RTs on all of the trials in the pure block to the non-cued trials in the mixed blocks (i.e., 0 ms SOA trials). A mixed-effects ANOVA assessing the effects of group and condition revealed a main effect of condition (*F*(1, 90) = 177.627, *p* < 0.001), whereby participants responded significantly faster on non-cued trials in the pure block compared to the mixed block (Table 3). No main effect of group (*F*(3,90) = 0.792, *p* = 0.501) or interaction between group and condition (*F*(3, 90) = 1.535, *p* = 0.211) was observed.

#### 3.2.2 Warning benefit

A mixed-effects ANOVA was conducted to assess the effects of and interaction between SOA (0 ms, 100 ms, 300 ms, 500 ms) and group on proactive inhibition. This revealed a significant effect of SOA^1^ (*F*(2.431, 218.776)=162.919, *p*<0.001), but no significant effect of group (*F*(3, 90) = 1.823, *p* = 0.149) nor group by SOA interaction (*F*(7.293, 218.776) = 1.074, *p* = 0.382). Post-hoc *t*-tests revealed significant differences between all SOAs, with fastest RTs observed at 500 ms SOAs, and slowest at 0 ms SOA (i.e., non-cued trials).

#### 3.2.3 Effects of anxiety and intolerance of uncertainty on proactive inhibition

The main effect of SOA on RT was unaffected after inclusion of covariates for both preparation cost and warning benefit, however, a main effect of group on warning benefit emerged after covarying for intolerance of uncertainty (IUS Total: *F*(3, 89) = 3.130, *p* = 0.030; IUS Self-Implications subscale: *F*(3, 89) = 2.911, *p* = 0.039). Post-hoc *t*-tests comparing RTs between groups (in the absence of covariates) revealed these group differences to be predominantly due to greater RTs in the AN group compared to HCs at all SOAs in the mixed block but not the pure block, though these findings did not survive correction for multiple comparisons. This suggest that group differences in proactive inhibition exist between AN and HC, but are masked by individual differences in intolerance of uncertainty.

Uncorrected exploratory correlational analyses exploring the relationship between proactive inhibition outcomes on the cued RT task and intolerance of uncertainty (IUS Total and subscale scores) across all of the groups revealed a positive association between intolerance of uncertainty and preparation cost (IUS Self-Implications subscale: *r*^*s*^ = 0.217, *p* = 0.036), but no other relationships were observed (all *r*^*s*^ ≥ 0.156, *p* ≥ 0.134). However, these findings did not survive Bonferroni correction for multiple comparisons.

### 3.3 Stop signal task

#### 3.3.1 Strategic proactive inhibition assessed by manipulating uncertainty across task blocks

##### 3.3.1.1 Effect of stop probability

Group differences in strategic proactive inhibition were assessed using a block-wise manipulation, comparing performance across four blocks of the task that varied in the proportion of stop trials (0%, 15%, 25%, 35%).

Mean RT on go trials (*χ*^2^(3) = 97.690, *p* < 0.001) and stop accuracy (*F*(2166) = 22.672, *p* < 0.001) increased with the degree of stop signal probability, whereas go accuracy decreased (trend: *χ*^2^(3) = 6.957, *p* = 0.073). Participants reacted more quickly and with greater go accuracy during the block with no stop trials compared to blocks during which stop trials occurred, and showed the greatest RTs and stop accuracy during the block with the greatest stop trials. Post-error slowing also increased as stop signal probability increased, though this was not significant (*F*(2166) = 0.653, *p*=0.522).

##### 3.3.1.2 Main effects of group and group-by-block interactions

The AN group consistently showed the slowest RTs on go trials and the greatest stop accuracy, whereas the BED group showed the fastest RTs (see Supplement D for a visual illustration).

As the distribution of mean RT and go accuracy data could not be normalised, Kruskal-Wallis tests were conducted to explore the main effect of group separately for each block. These did not find any significant effect of group on mean RT on any of the blocks (0% stop trials: *χ*^2^(3) = 4.440, *p* = 0.218; 15% stop trials: *χ*^2^(3) = 2.511, *p* = 0.473; 25% stop trials: *χ*^2^(3) = 3.152, *p* = 0.369; 35% stop trials: *χ*^2^(3) = 5.049, *p* = 0.168) or on go accuracy on any of the blocks (0% stop trials: *χ*^2^(3) = 5.459, *p* = 0.141; 15% stop trials: *χ*^2^(3) = 2.980, *p* = 0.395; 25% stop trials: *χ*^2^(3) = 2.707, *p* = 0.439; 35% stop trials: *χ*^2^(3) = 2.972, *p* = 0.396).

Mixed-design ANOVAs similarly revealed no effect of group on stop accuracy (*F*(3,83) = 1.312, *p* = 0.276) or post-error slowing (*F*(3,83) = 0.893, *p* = 0.449), or interactions between group and block on either measure (stop accuracy: *F*(6,166) = 1.028, *p* = 0.409; post-error slowing: *F*(6,166) = 0.726, *p* = 0.629).

##### 3.3.1.3 Controlling for confounding effects of intolerance of uncertainty on proactive inhibition

The main effect of block on stop accuracy reached trend-level significance after controlling for intolerance of uncertainty (IUS Total: *F*(2164)=3.016, *p*=0.052; IUS Self-Implications subscale scores: *F*(2, 164) = 2.886, *p* = 0.059), suggesting that intolerance of uncertainty contributed substantially to the variability of stop accuracy across participants, reducing the sensitivity of our initial analysis.

Post-hoc exploratory correlational analysis did not reveal any relationships between proactive inhibition outcomes on the SST and intolerance of uncertainty (all *r*^*s*^> −0.154, *p* ≥ 0.155).

#### 3.3.2 Reactive inhibition

Reactive inhibition was assessed from mean RT on go trials and the SSRT (mean SSD subtracted from the go RT that corresponded to the participant's inhibition accuracy). As many participants did not converge at 50% inhibition accuracy as intended by the task, the mean SSD and inhibition (stop) accuracy (%) were analysed as secondary outcome measures to aid interpretation.

Group differences in reactive inhibition were assessed using a single block on the SST that included 75% go trials and 25% stop trials. The groups did not differ statistically in any of the outcome measures (SSRT: *F*(3,89) = 1.609, *p* = 0.193; stop accuracy: *F*(3,89) = 1.355, *p* = 0.262; mean RT: χ^2^(3) = 3.152, *p* = 0.369; mean SSD: *χ*^2^(3) = 4.820, *p* = 0.185). However, visual inspection of the group differences did suggest that the (non-significant) differences were in the direction we had predicted, i.e., the AN group showed the greatest stop accuracies, mean stop signal delays and mean RTs (suggesting a more inhibited approach), whereas the BN group showed the poorest accuracies, smallest stop signal delays and fastest RTs (Table 4). Surprisingly though, the AN group showed the largest SSRT (thought to index greater impulsivity/poorer inhibitory control), whereas the BN group showed the smallest SSRTs.

**Table 4:** Means (SD) for each stop signal task (SST) outcome measure in each group.

	HC	AN	BN	BED
SSRT	558.15 (297.551)	628.89 (322.635)	512.88 (255.743)	517.64 (289.703)
Mean RT (ms)	549.84 (228.227)	614.60 (261.978)	525.00 (210.829)	531.55 (256.044)
Mean SSD	272.72 (186.502)	300.42 (192.440)	245.83 (156.607)	264.57 (196.490)
Stop Accuracy (%)	48.1% (15.1%)	50.7% (14.9%)	43.3% (16.2%)	42.7% (14.2%)

##### 3.3.2.1 Controlling for confounding effects of anxiety and intolerance of uncertainty on proactive inhibition

Including self-reported intolerance of uncertainty (total/subscale scores) as a covariate did not affect the initial observations: no group differences were observed on measures of reactive inhibition (stop accuracy (%), log-transformed SSRT).

### 3.4 Questionnaire outcomes

Self-reported impulsivity was explored using the BIS-11 and the behavioural inhibition subscale (BIS) of the BIS-BAS questionnaire.

As some of the subscales of the BIS-11 and the BIS subscale were non-normally distributed, Kruskal-Wallis analyses were conducted to explore differences between group means. These revealed a significant difference between groups on the BIS subscale of the BIS-BAS, the BIS-11 Attention and Motor subscale scores and BIS-11 total scores, whereas no group differences were observed on the BIS-11 Non-planning subscale (Table 5). Post-hoc Mann-Whitney *U* tests revealed that while the AN and BN groups reported greater impulsivity in some domains compared to HC (BIS-11 Attention (both AN and BN), BIS (AN only) and BIS-11 Total (BN only)), the BN group reported greater overall impulsivity compared to the AN group (BIS-11 Total) and the AN group reported less motor impulsivity (BIS-11 Motor) compared to all other groups. No other comparisons remained significant after correction for multiple comparisons.

**Table 5:** Mean (SD) scores on self-reported measures of behavioural inhibitory control and statistical comparisons between groups.

	Means (SD)				Kruskal-Wallis	Post-hoc Mann-Whitney *U* tests					
	HC	AN	BN	BED		HC vs. AN	HC vs. BN	HC vs. BED	AN vs. BN	AN vs. BED	BN vs. BED
BIS-11 Total	60.26 (10.242)	61.85 (9.871)	69.18 (11.702)	67.91 (13.487)	χ^2^(3) = 9.683, *p* = 0.021	*U* = 361.00, *z* = −0.061, *p* = 0.952	*U* = 201.50, *z* = −2.493, *p* = 0.013	*U* = 100.00, *z* = −1.562, *p* = 0.118	*U* = 192.50, *z* = −2.658, *p* = 0.008*	*U* = 103.00, *z* = −1.466, *p* = 0.143	*U* = 132.50, *z* = −0.172, *p* = 0.864
BIS-11 Attention	14.70 (4.672)	18.89 (3.856)	19.40 (4.387)	19.27 (4.756)	χ^2^(3) = 15.079, *p* = 0.002	*U* = 181.50, *z*= −3.175, *p*=0.001*	*U* = 157.50, *z* =−3.307, *p*=0.001*	*U* = 72.50, *z* = −2.455, *p* = 0.014	*U* = 322.50, *z* = −0.276, *p* = 0.783	*U* = 143.00, *z* = −0.178, *p* = 0.859	*U* = 130.50, *z* = −0.241, *p* = 0.809
BIS-11 Motor	22.00 (3.541)	19.00 (4.674)	23.26 (4.776)	25.27 (5.985)	χ^2^(3) = 16.141, *p* = 0.001	*U* = 205.50, *z* = −2.764, *p* = 0.006*	*U* = 292.50, *z* = −0.828, *p* = 0.407	*U* = 97.00, *z* = −1.667, *p* = 0.095	*U* = 163.00, *z* = −3.211, *p* = 0.001*	*U* = 58.00, *z* = −2.925, *p* = 0.003*	*U* = 108.00, *z* = −1.018, *p* = 0.309
BIS-11 Non-planning	12.00 (2.787)	12.89 (3.786)	13.60 (3.416)	12.73 (4.101)	χ^2^(3) = 3.473, *p* = 0.324	*U* = 322.50, *z* = −0.732, *p* = 0.464	*U* = 239.00, *z* = −1.814, *p* = 0.070	*U* = 139.00, *z* = −0.309, *p* = 0.757	*U* = 279.00, *z* = −1.079, *p* = 0.281	*U* = 139.50, *z* = −0.292, *p* = 0.770	*U* = 106.00, *z* = −1.092, *p* = 0.275
BIS-BAS BIS subscale	21.70 (3.517)	25.78 (1.672)	24.16 (2.925)	24.45 (3.387)	χ^2^(3) = 19.059, *p* < 0.001	*U* = 120.50, *z* = −4.258, *p* < 0.001*	*U* = 202.00, *z* = −2.493, *p* = 0.013	*U* = 83.00, *z* = −2.116, *p* = 0.034	*U* = 230.50, *z* = −1.990, *p* = 0.047	*U* = 125.00, *z* = −0.770, *p* = 0.441	*U* = 125.00, *z* = −0.433, *p* = 0.665

* *p* < 0.05 after Bonferroni correction. Uncorrected *p*-values are presented in the table.

### 3.5 Correlations between task-based and clinical variables

Reactive inhibition outcomes showed negative associations with BMI (SSRT, mean RT [trend]) and binge frequency (SSRT [trend]), but no other relationships were observed (Table 6). This indicates that individuals with lower BMIs showed longer RTs on go trials. In contrast to what was predicted, lower BMI and reduced frequency of binge eating episodes were associated with greater SSRTs.

**Table 6:** Spearman's correlation coefficients describing the relationship between clinical variables and task-based measures of inhibitory control across the ED groups.

			**Proactive inhibition**				**Reactive inhibition**	
	**(Simple cued RT)**		**(Stop signal task)**				**(Stop signal task)**	
	**Preparation cost**	**Warning benefit**	**Go accuracy change**	**Mean RT change**	**Stop accuracy change**	**Post-error slowing change**	**SSRT**	**Mean RT**
Length of illness	*r_s_* = 0.151, *p* = 0.227	*r_s_* = −0.106, *p* = 0.395	*r_s_* = −0.314, *p* = 0.014	*r_s_* = 0.027, *p* = 0.839	*r_s_* = −0.110, *p* = 0.399	*r_s_* = 0.052, *p* = 0.689	*r_s_* = 0.174, *p* = 0.174	*r_s_* = 0.186, *p* = 0.145
EDE-Q Global	*r* = −0.040, *p* = 0.747	*r* = 0.043, *p* = 0.730	*r_s_* = −0.211, *p* = 0.102	*r_s_* = −0.055, *p* = 0.673	*r_s_* = −0.146, *p* = 0.262	*r_s_* = 0.165, *p* = 0.204	*r_s_* = −0.014, *p* = 0.914	*r_s_* = 0.019, *p* = 0.880
Binge frequency	*r* = −0.041, *p* = 0.744	*r* = −0.047, *p* = 0.706	*r_s_* = 0.052, *p* = 0.690	*r_s_* = −0.065, *p* = 0.620	*r_s_* = −0.122, *p* = 0.348	*r_s_* = 0.014, *p* = 0.914	*r_s_* = −0.232, *p* = 0.067	*r_s_* = −0.168, *p* = 0.189
Purge frequency	*r_s_* = −0.055, *p* = 0.663	*r_s_* = −0.061, *p* = 0.629	*r_s_* = −0.066, *p* = 0.614	*r_s_* = 0.025, *p* = 0.850	*r* = 0.193, *p* = 0.136	*r* = −0.271, *p* = 0.034	*r_s_* = −0.134, *p* = 0.294	*r_s_* = −0.101, *p* = 0.433
BMI	*r_s_* = −0.074, *p* = 0.481	*r_s_* = 0.075, *p* = 0.470	*r_s_* = 0.010, *p* = 0.928	*r_s_* = −0.205, *p* = 0.057	*r_s_* = 0.029, *p* = 0.791	*r_s_* = 0.061, *p* = 0.575	*r_s_* = −0.218, *p* = 0.039	*r_s_* = −0.191, *p* = 0.071

Proactive inhibition outcomes also showed a relationship with clinical variables across the ED groups, with go accuracy change negatively associated with length of illness and change in post-error slowing negatively associated with purge frequency. This suggests that individuals reporting a longer illness duration and more frequent purging episodes show less adjustment of their motor responses as uncertainty changes, suggesting poorer proactive inhibition. However, none of these findings survived correction for multiple comparisons, therefore the findings must be interpreted with caution.

## 4 Discussion

This study investigated whether proactive and reactive motor inhibitory control differed across ED. The self-report data indicated that individuals with BN considered themselves to have less overall inhibitory control compared to those with AN and HCs, whereas individuals with AN reported the greatest motor inhibitory control compared to all other groups (see Section 3.4). In terms of behavioural performance on the tasks, the data did not reveal any group differences in reactive inhibitory control. However, individuals with AN showed greater proactive inhibition compared to HC, and this appears to be related to individual differences in intolerance of uncertainty. Therefore specific elements of inhibitory control appear to be different in different ED. Overall, the data provide limited support for a spectrum model of EDs based on motor inhibitory control, particularly with regards to perceived (self-reported) inhibitory control, but suggest that the different subtypes of motor inhibitory control (proactive/reactive) may be differentially implicated within each of the EDs.

We did not observe any differences in reactive inhibitory control between the groups. This is in contrast to the literature supporting impaired reactive inhibitory control in individuals with binge-type eating disorders (for reviews, see Bartholdy et al., 2016c; Wu et al., 2013b), however such findings have not been consistently reported in the literature (Boisseau et al., 2012, Claes et al., 2006). These findings suggest that reactive motor inhibitory control to stimuli that is not disorder-specific may not be impaired in binge-type EDs, and therefore seems unlikely to be a general motor deficit contributing to ED psychopathology. In contrast to that hypothesised in spectrum models of EDs (Brooks, 2016, Brooks et al., 2012), the AN group showed the largest SSRTs (thought to reflect poorer reactive inhibitory control), although was not significantly different from the other groups, and poorer inhibitory control on the SST in AN has been reported previously (Galimberti et al., 2012). The discrepancy between our findings and those reported by previous studies may be due to the task and computational methods used. Typically, versions of the SST that make use of the tracking method (as in the present study) dynamically adjust the SSD to enable 50% inhibition accuracy: in this context, the SSRT can be calculated by subtracting the mean SSD from the mean RT on correct go trials (Logan, 1994, Verbruggen et al., 2008). However, if convergence at 50% inhibition accuracy is not achieved, this conventional method of SSRT calculation is not suitable (for review, see Verbruggen and Logan, 2008). In the present study, convergence at ~50% correct inhibition for all blocks was only achieved by a small proportion of each group, which may have been due to either the small number of trials per block, or the manipulation of the probability of a stop trial occurring. We therefore used an alternative method for calculating the SSRT, which has been used previously (Bartholdy et al., 2016b, Nederkoorn et al., 2012). Unfortunately, our findings question the sensitivity of this alternative method of SSRT calculation as an index of impulsivity: as in the present study, SSRTs appeared to be largely driven by the relatively longer mean RTs and greater stop accuracy in the AN group. In other words, if the go RT used to calculate the SSRT is determined by the stop accuracy, then in the context of similar RTs, a greater stop accuracy will result in greater SSRTs. Similarly, in the context of similar stop accuracies, greater RTs will result in greater SSRTs. However, greater RT and greater stop accuracies can be argued to reflect greater inhibitory control, so do not reflect what would typically be considered as impulsive behaviour. Thus, for cases whereby convergence at 50% stop accuracy has not been reached, it may be more beneficial to consider all behavioural outcomes together and interpret performance on one measure in the context of other behavioural indices, rather than relying on one outcome measure.

Our findings are consistent with the proposal that proactive inhibition is involved in ED pathology (Bartholdy et al., 2016a). Inspection of the outcome measures of two discrete computer tasks assessing proactive inhibition suggests the AN group tend to favour a more cautious approach in their responding compared to the other groups, i.e., showing slower mean RTs in every condition of both tasks and the greatest stop accuracy on the SST. This suggests individuals with AN prioritise accuracy over speed and may reflect greater tonic proactive inhibition. This is in accord with reports that individuals with AN show more perfectionistic behaviours (e.g., longer times checking their work; Lloyd et al., 2014) and attribute increased importance to decision accuracy (Sternheim et al., 2011b). Moreover, after controlling for individual differences in intolerance of uncertainty, we found that proactive inhibition (on the cued RT task) differed between the groups: this was driven by differences in the effect of warning cues on the cued RT task between the AN and HC groups. The AN group showed a greater preparation cost (i.e., greater slowing of responses during non-cued trials in the mixed block compared to the pure block) as they demonstrated longer RTs at all SOAs during the mixed block compared to the HC group, but no differences in RT in the pure block. This suggests that, relative to HCs, the AN group engage in greater proactive slowing in the context of uncertainty. Arguably, these findings may simply reflect generally slower behavioural responding, though there is little evidence to suggest that AN is associated with a general slowing of motor responses on such tasks (see Bartholdy et al., 2016c for review). It can be speculated that exaggerated proactive inhibition may contribute to the inhibited temperament thought to be characteristic of AN: i.e., contexts of uncertainty or symptom provocation (i.e., in the presence of food) may increase proactive inhibition, which may promote more inhibited, avoidant and/or restrictive behaviours as a means of dealing with the anxiety-provoking situation, and may impede the potential efficacy of exposure treatments for AN (Koskina et al., 2013).

Based on the evidence implying the involvement of altered inhibitory control in EDs, behavioural interventions have emerged that aim to manipulate inhibitory control as a means of improving ED symptoms (for reviews, see Bartholdy et al., 2016c; Stice et al., 2016; Turton et al., 2016). The present findings suggest that proactive inhibition may be a target for behavioural interventions for AN, and future studies should explore whether interventions that reduce anxiety related to uncertainty have any ancillary benefits on proactive inhibition. In contrast, the lack of any group differences in reactive inhibition suggests that behavioural paradigms may not wish to focus on reactive inhibition in a general context, though future studies may wish to further explore the degree to which disorder-relevant stimuli affects reactive inhibition specifically to determine its utility as a target of manipulation in such behavioural interventions.

This study has some limitations. The sample size of the BED group was small, and thus may not be a representative sample of the BED population. Moreover, this group has a comparably low average BMI (but high standard deviation) for this disorder, possibly due to the relatively short illness duration. This may have contributed to the lack of statistically significant results in the behavioural tasks, as a trend towards a positive relationship between BMI and SSRT has previously been reported in obese participants with and without BED (Mole et al., 2015), and differences in SSRT across different weight categories have been reported (see Bartholdy et al., 2016c for review). However, such a relationship was not observed in the present study (when all groups were considered together), thus the influence of BMI, if any, may be small. Moreover, while some studies have found evidence of poor motor inhibitory control in BED (Mole et al., 2015, Svaldi et al., 2014) this is not always the case (Wu et al., 2013a). In addition to limitations regarding sample size, there are limits with regards to generalisability of the findings as all the participants were women. This was specified in our inclusion criteria to reflect the relatively greater prevalence of EDs in women. However, a meta-analysis of healthy adults did not observe any sex differences in tasks involving inhibitory control, such as the SST (Cross et al., 2011), though sex differences in proactive inhibition have not yet been explored.

This study is the first to explicitly explore proactive inhibition in EDs using multiple neurocognitive methods for assessing proactive inhibition, and provides early empirical support for altered proactive inhibition as a correlate of AN. Our findings suggest that motor inhibitory control is not uniformly implicated across EDs. Studies of proactive inhibition in larger samples (particularly in the BED group) are required to provide more definitive evidence on the involvement of proactive and reactive inhibition in EDs.

## Conflicts of interest

The authors report no conflicts of interest

## Footnotes

1 Due to violation of the assumption of sphericity, Huynh Feldt degrees of freedom and *p* values are reported.
