# Supplementary material for: Proactive and reactive inhibitory control in eating disorders
Source: Psychiatry Res. 2017 Sep;255:432–40. doi: 10.1016/j.psychres.2017.06.073 (PMC5555256; doi:10.1016/j.psychres.2017.06.073)
Supplement: Supplementary file 1 — Supplementary material [file mmc1.docx]

# Supplement A. Schematic diagram of the cued RT task


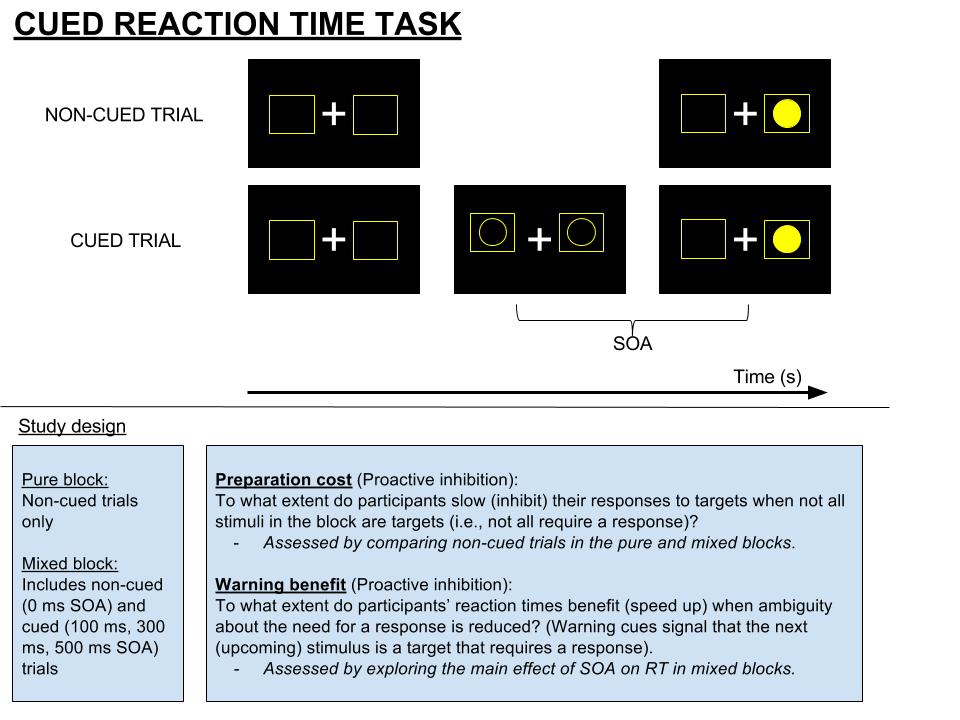


Note: SOA = stimulus onset asynchrony; RT = reaction time; s = seconds; ms = milliseconds
