# Supplementary figures and images for: Proactive and reactive inhibitory control in eating disorders
Source: Psychiatry Res. 2017 Sep;255:432–40. doi: 10.1016/j.psychres.2017.06.073 (PMC5555256; doi:10.1016/j.psychres.2017.06.073)

# Supplement B. Schematic diagram of the stop signal task


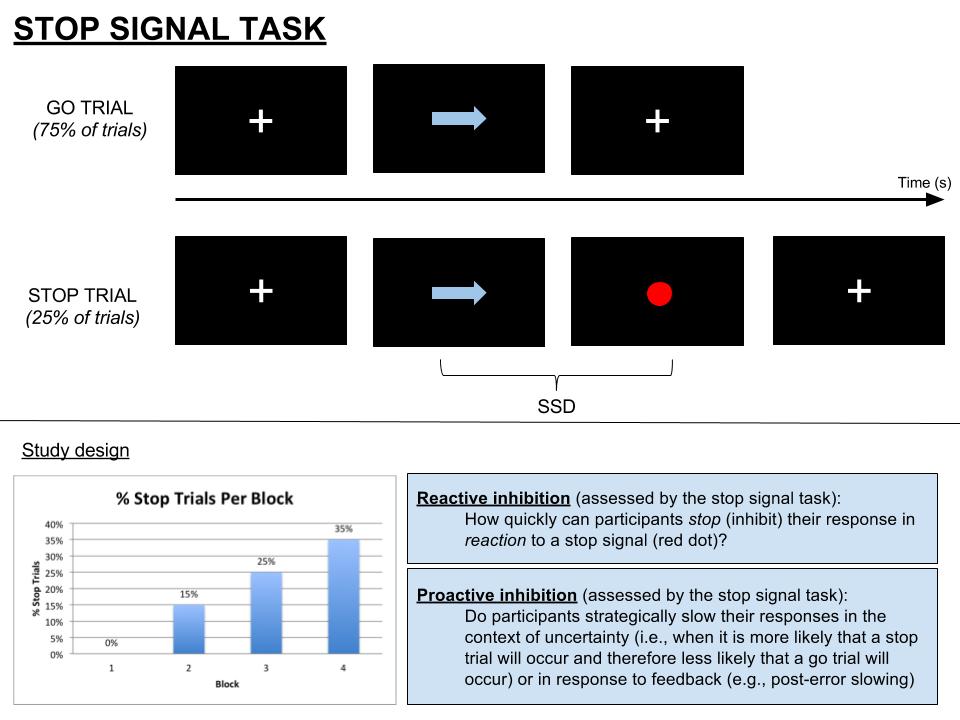


Note: SSD = stop signal delay; s = seconds

Supplement: Supplementary file 2 — Supplementary material [file mmc2.docx]
