# Supplementary material for: Proactive and reactive inhibitory control in eating disorders
Source: Psychiatry Res. 2017 Sep;255:432–40. doi: 10.1016/j.psychres.2017.06.073 (PMC5555256; doi:10.1016/j.psychres.2017.06.073)
Supplement: Supplementary file 3 — Supplementary material [file mmc3.docx]

# Supplement C. Summary of post-hoc statistical tests

## Demographic information

### EDE-Q Global scores (main effect of group)

Unsurprisingly, the difference between groups in global ED pathology was driven by lower ED pathology in the HC group compared to all other groups (AN: *U*=13.0, *z*=-6.211, *p*<0.001; BN *U*=5.0, *z*=-6.281, *p*<0.001; BED: *U*=3.0, *z*=--4.714, *p*<0.001). No differences in ED pathology were observed between the ED groups (all *U*≥106.0, *z*≥1.368, *p*≥0.171).

### BMI (main effect of group)

Group differences in BMI were driven by lower BMI in the AN group compared to all other groups (BN: *t*(53)=-7.550, *p*<0.001; BED: *t*(10.887)=-5.667, *p*<0.001; and HC: *t*(54)=-9.047, *p*<0.001), and higher BMI in the BED group compared with the HC group: *t*(10.681)=-3.218, *p*<0.008).

### Illness duration (main effect of group)

The ED groups differed in illness duration, with the greatest duration reported by the AN group and the shortest reported by the BED group (AN vs. BN: *U*=215.5, *z*=-2.745, *p*=0.006; AN vs. BED: *U*=51.0, *z*=-3.231, *p*=0.001; BN vs. BED (trend): *U*=95.5, *z*=-1.724, *p*=0.085).

## Cued RT task

### Warning benefit (main effect of SOA)

Post-hoc *t*-tests revealed significant differences between all SOAs: significantly faster RTs were observed at 500 ms SOAs compared to 0 ms (*t*(93)=17.766, *p*<0.001), 100 ms ((*t*(93)=13.722, *p*<0.001) and 300 ms ((*t*(93)=17.483, *p*<0.001). Participants also responded more quickly at 100 ms (*t*(93)=9.521, *p*<0.001) and 300 ms (*t*(93)=5.233, *p*<0.001) compared to the non-cued (0 ms) trials, however participants responded faster at 100 ms compared to 300 ms SOA (*t*(93)=-8.167, *p*<0.001).

### Effects of anxiety and intolerance of uncertainty on proactive inhibition (main effect of group)

Post-hoc *t*-tests (not including covariates) comparing RTs between groups the group differences that emerged after covarying for anxiety and intolerance of uncertainty to be driven by greater RTs in the AN group compared to HCs at all SOAs in the mixed block (0 ms: *t*(42.541)=-1.748, *p*=0.088; 100 ms: *t*(44.410)=-2.196, *p*=0.033; 300 ms: *t*(42.177)=-2.531, *p*=0.015; 500 ms: *t*(42.278)=-2.273, *p*=0.028) but not the pure block (0 ms: *t*(52)=-1.038, *p*=0.304), though these findings did not survive correction for multiple comparisons.

## Stop signal task

#### Strategic proactive inhibition: the effect of stop probability (main effect of block)

Participants reacted more quickly during the block with no stop trials compared to blocks during which stop trials occurred (0% vs. 15% stop trials: *z*=-5.595, *p*<0.001; 0% vs. 25% stop trials: *z*=-6.083, *p*<0.001; 0% vs. 35% stop trials: *z*=-7.119, *p*<0.001) and showed the greatest RTs during the block with the greatest stop trials (15% vs. 35% stop trials: *z*=-6.023, *p*<0.001; 25% vs. 35% stop trials: *z*=-3.822, *p*<0.001). A difference in mean RT was observed the blocks using 15% and 25% stop trials (*z*=-1.981, *p*=0.048), however this did not survive multiple comparisons correction. Differences in stop accuracy were driven by lower accuracy in the 15% stop block compared to both the 25% stop block (*t*(86)=-3.692, *p*<0.001) and the 35% stop block (*t*(86)=-6.572, *p*<0.001), and poorer accuracy in the 25% compared to 35% stop block persisting after Bonferroni correction (*t*(86)=-3.056, *p*=0.003). Post-error slowing also increased as stop signal probability increased, though this was not significant (*F*(2,166)=0.653, *p*=0.522).

Go accuracy decreased with the degree of stop signal probability (trend: *χ*^2^(3)=6.957, *p*=0.073). This was driven by greater accuracy in the block with no stop trials compared to the block with the greatest stop probability (0% vs. 35%: z=-2.773, *p*=0.006) and a trend towards greater accuracy compared to the 25% stop block (*z*=-1.831, *p*=0.067), though this latter comparison did not remain after correction for multiple comparisons. Go accuracy did not differ between the 0% and 15% stop blocks (*z*=-1.587, *p*=0.113), or between any other blocks (15% vs. 25%: *z*=-1.375, *p*=0.169; 15% vs. 35%: *z*=-.853, *p*=0.394; 25% vs. 35%: *z*=-1.196, *p*=0.232).
