# Supplementary material for: Proactive and reactive inhibitory control in eating disorders
Source: Psychiatry Res. 2017 Sep;255:432–40. doi: 10.1016/j.psychres.2017.06.073 (PMC5555256; doi:10.1016/j.psychres.2017.06.073)
Supplement: Supplementary file 4 — Supplementary material [file mmc4.docx]

# Supplement D.


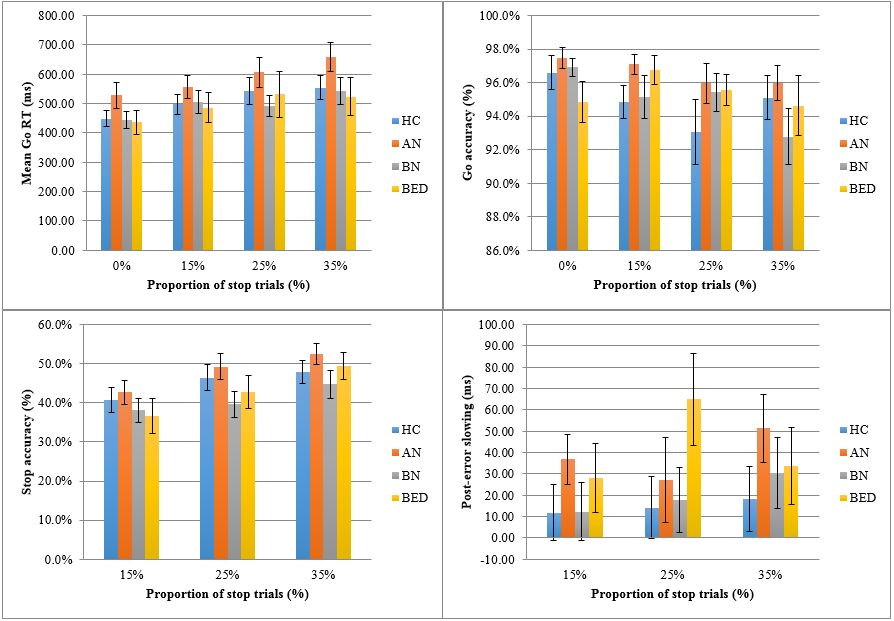


Individual group means for proactive inhibition outcomes on the stop signal task across blocks containing differing proportions of stop trials. Clockwise from top left: Group averages for go mean RT (ms), go accuracy (%), average, and stop accuracy (%), and post-error slowing of reaction times on go trials following a stop signal (ms) across the 4 blocks of the stop signal task. Error bars denote standard error.
